# Fucoidans of Moroccan Brown Seaweed as Elicitors of Natural Defenses in Date Palm Roots

**DOI:** 10.3390/md18120596

**Published:** 2020-11-26

**Authors:** Soukaina Bouissil, Zainab El Alaoui-Talibi, Guillaume Pierre, Halima Rchid, Philippe Michaud, Cédric Delattre, Cherkaoui El Modafar

**Affiliations:** 1 Laboratoire d’Agrobiotechnologie et Bioingénierie, Faculté des Sciences et Techniques Marrakech, Université Cadi Ayyad, Marrakesh 40000, Morocco; soukaina.BOUISSIL@etu.uca.fr (S.B.); Z.elalaouitalibi@uca.ma (Z.E.A.-T.); elmodafar@uca.ac.ma (C.E.M.); 2 Institut Pascal, Université Clermont Auvergne, CNRS, SIGMA Clermont, F-63000 Clermont-Ferrand, France; guillaume.pierre@uca.fr (G.P.); philippe.michaud@uca.fr (P.M.); 3 Laboratoire de Biotechnologies et Valorisation des Ressources Végétales, Faculté des Sciences, Université Chouaib Doukkali, El Jadida 24000, Morocco; rchid.h@ucd.ac.ma; 4 Institut Universitaire de France (IUF), 1 Rue Descartes, 75005 Paris, France

**Keywords:** sulfated polysaccharides, natural defenses, phenolic metabolism, phenylalanine ammonia-lyase

## Abstract

Fucoidans from Moroccan brown seaweed *Bifurcaria bifurcata* and *Fucus spiralis* were tested for their elicitor activity after their purification and complete characterization. The fucoidans of *B. bifurcata* (BBF) and of *F. spiralis* (FSF) were extracted and purified then characterized by infrared spectroscopy, proton nuclear magnetic resonance spectroscopy and size exclusion chromatography. The results show that BBF and FSF are mainly sulfated with 45.49 and 49.53% (*w*/*w*) sulfate, respectively. Analysis of neutral sugars determined by gas chromatography–mass spectrometry showed that FSF and BBF were mainly composed of 64% and 91% fucose and 20% and 6% galactose, respectively, with a few other sugars such as glucose (8% in FSF), rhamnose (1% in BBF) and mannose (8% in FSF and, 2% in BBF). The eliciting activity of these sulfated polysaccharides in stimulating the natural defenses of the date palm was evaluated through the activity of phenylalanine ammonia-lyase (PAL), and the increase in phenols and lignin content in the roots. The results obtained clearly show that the two fucoidans early and intensely stimulate the natural defenses of the date palm after 24 h of treatments. This remarkable elicitor effect seems to be linked to the sulfated groups compared to non-sulfate alginates extracted from the same algae. These results open promising perspectives for a biological control approach against date palm diseases.

## 1. Introduction

Sulfated polysaccharides are increasingly recognized for their broad spectrum of biological activities. They usually found in large quantities in brown seaweeds. In addition, the polysaccharides structures depend on the algae species. Thus, various biological activities could be discovered with each new sulfated polysaccharide extraction [1].

Amongst these polysaccharides, the most studied was carrageenan [2], ulvan [3] and fucoidans. Fucoidans at the molecular level constitute a polymer of L-fucose linked by (1,3) and (1,4) with residues mainly sulfated on C-4 [4,5]. The characteristic structure of fucoidans rich in L-fucose and sulfated ester groups has generated widespread interest due to their therapeutic effects. Several works reported the biological proprieties of fucoidans [6], namely antioxidant [7,8], antitumor [9,10,11,12] and anticoagulant [13]. In addition to these biological applications, fucoidans and their oligosaccharides have also been the subject of other studies on biostimulants of defence mechanisms in plants [14,15]. They were also suggested as biological approaches to control plants disease [16], by benefiting from their stimulating effect of early and late defensive responses. It is reported that biological and biostimulant properties of fucoidans depend in particular on the degree of sulfation [8] and on their various physico–chemical properties [5].

The aim of this work is to study the potential activity of fucoidans to elicit the natural defence mechanisms in date palm (*Phoenix dactylifera* L.) roots as a monocotyledon plant. Through an innovative elicitation model allowing the treatment of the roots which are the site of infection of the date palm by *Fusarium oxysporum* f. sp. *albedinis* (Foa), a telluric pathogen causing the fatal disease (Bayoud) of date palm [17]. In response to Bayoud disease, date palm develops numerous defence mechanisms in roots such as the induction of phytoalexins [18], the accumulation of caffeoyl shikimic acids [19,20] and the reinforcement of the cell walls by lignin and phenolic compounds [21]. These defence mechanisms all depend on, phenylalanine ammonia-lyase (PAL) activity, triggering the phenlypropanoid pathway [17]. The activity of this enzyme governs the defence mechanisms induced in sensitive and resistant varieties during a date palm and Foa interactions [22]. In this context crude fucoidans, extracted from two brown algae *Bifurcaria bifurcata* and *Fucus spiralis* from the Atlantic coast of Morocco were structurally characterized and tested for their possible eliciting effect on the defence mechanisms of the date palm roots.

## 2. Results and Discussion

### 2.1. Chemical Composition of Crude Fucoidans

The abundance of the two brown algae *B. bifurcata* and *F. spiralis* on the Moroccan Atlantic coast was the reason for the choice of these two species. The yield as well as the chemical composition of the fucoidans extracted from these species are shown in Table 1. FSF and BBF yields were around 8 and 2%, respectively, based on algae dry weight.

Colorimetric assays show that FSF and BBF contained principally neutral sugar from 45.23 to 51.16 for *B. bifurcata* (BBF) and of *F. spiralis* (FSF), respectively, those extracted fucoidans were also highly sulfated (FSF, 49.53% and BBF, 45.49%).

The main neutral sugars which constitute FSF and BBF was determined by gas chromatography–mass spectrometry (GC-MS) analysis and the result reported in Table 2 shows that was the L-fucose with 63.98 and 90.68%, respectively, based on dry weight of sulfated polysaccharides (FSF and BBF).

The results obtained (Table 1) are different from those demonstrated for fucoidans of *B. bifurcata* from Britain with a higher extraction yield (17% *w*/*w*), 40–42% of carbohydrate with 22.2% of sulfates [23]. Compared to fucoidans extracted from other green algae, the BBF extraction yield remained much lower than that registered of fucoidans of *Cystoseira compressa* (5.2% *w*/*w*) [24] and *Cystoseira barbata* (5.45% *w*/*w*) [8], whereas, it is close to 2.8% of fucoidans purified from *C. crinite* and to 2.2% extracted from *Dictyota dichotoma* [25,26], while the FSF yield was more important than that reported for the algae mentioned above. On the other hand, the sulfates concentration of extracted polysaccharides (FSF and BBF) was much higher than those reported for fucoidans extracted from *Cystoseira* and *Sargassum* species [8,24,27]. Elsewhere, fucoidans from *Alaria* sp. and *Saccharina japonica* at spore production was highly sulfated than fucoidans obtained at vegetative status of these brown algae species [28]. Thus, fucoidans’ yield and their overall chemical composition could be influenced by the procreating status of seaweed [29]. L-fucose was the principal constitutive monosaccharide of FSF and BBF, with a Fuc_p_/Gal_p_ ratio of 3.2 and 14.7, respectively (Table 2), indicating a predominate amount of L-fucose than galactose. This composition was similar to that found in fucoidans of *Saccharina cichorioides* (Fuc_p_/Gal_p_ ratio of 13.84) related to 88.6% mol of L-fucose and 6.4% mol of galactose [27]. The ratio found in this work for BBF (14.7) appeared higher than that recorded for fucoidans of *S. japonica* with Fuc_p_/Gal_p_ of 1.13 [27], and the ratio obtained from *Undaria pinnatifida* fucoidans which was equal to 1.39 [29]. More studies carried out on fucoidans reported the lower Fuc_p_/Gal_p_ ratio for fucoidans of *C. compressa* (2.57) [24], *C. barbata* (1.3) [8], *Agarum cribrosum* (2.63) [30], *Lachemilla angustata* (3.93) [31] and *Fucus evanescens* (8.2) [27]. The monosaccharide composition obtained for FSF and BBF reported the presence of more than 50% of L-fucose; this can explain the higher sulfate concentration in FSF and BBF. It was reported that concentration of sulfated residues depend on the nature of fucoidans monosaccharide composition [27,31].

### 2.2. Proton Nuclear Magnetic Resonan ce (^1^H-NMR) and Infrared (ATR-FTIR) Spectroscopies

**Figure 1:**
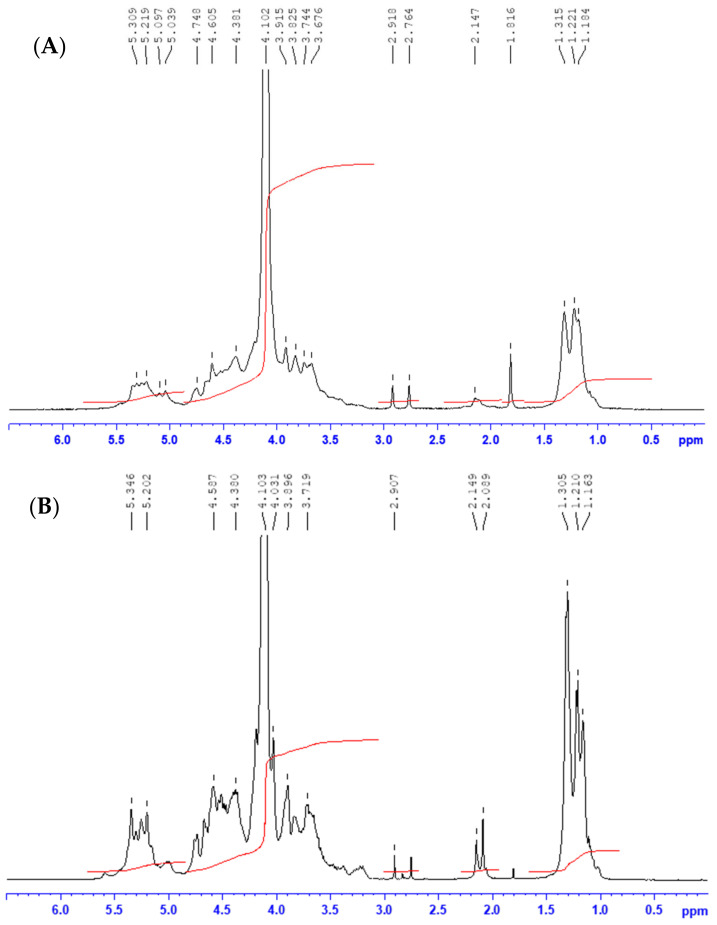
^1^H NMR spectra of sulfated polysaccharides from (**A**) *B. bifurcata* (BBF) and (**B**) *F. spiralis* (FSF) at 60 °C in D_2_O solution.

To better characterize the fucoidans, an ^1^H NMR analysis was carried out. FSF and BBF spectra are presented in Figure 1. The two spectra exhibit five regions characteristic of fucoidans. The intense peaks at 1.34 and at 1.22 ppm are from the H6 methyl protons of L-fucopyranose [32]. Signal at 2.14 ppm refer to the methyl protons of the O-acetyl groups [32]. The spectrum between 4.1 and 3.7 ppm corresponds to the protons of the ring (H2-H5) [8]. The signal around 4.3 ppm, is related to the protons of the 4-O-sulfated monosaccharides [33,34]. It is more intense in the case of FSF than BBF, which corroborates the slight difference in sulfates proportions between the two samples (Table 1). Finally the signals region between 5.3 and 5.03 ppm, are attributed to the C-H proton of substituted O=C and to proton H1 of monosaccharides-α-L-fucopyranose [32]. The spectra obtained for FSF and BBF are very similar to those obtained for fucoidans of *Fucus vesiculosus* and *Ascophyllum nodosum* [33], *C. barbata* [8] and *C. compressa* [24].

**Figure 2:**
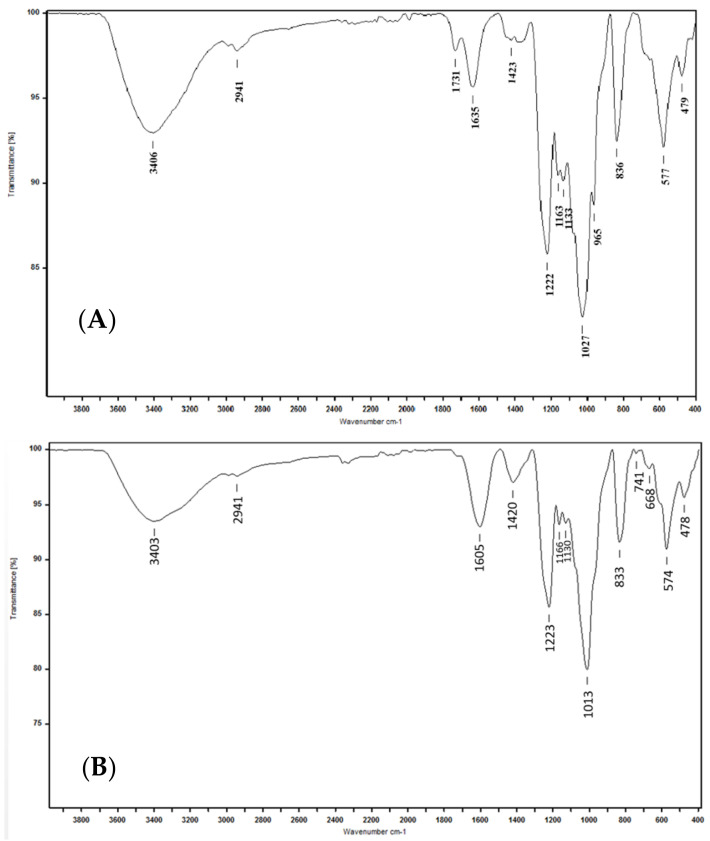
ATR-FTIR spectra of sulfated polysaccharides from (**A**) *B. bifurcata* (BBF) and (**B**) *F. spiralis* (FSF).

In parallel, an infrared analysis was carried out. The Attenuated Total Reflectance ATR-FTIR spectra of BBF and FSF were represented in Figure 2. The two spectra showed characteristics bands at 3406–3403, 2941, 1635–1605, 1423–1420, 1222–1223,1027–1013, 836–833, 577–574 and 479–748 cm^−1^ (Figure 2). The absorption peaks around 3406–3403 and 2941 cm^−1^ are attributed to the elongation of (O-H) and asymmetric vibrations of (C-H), respectively [35]. The signals around 1635 cm^−1^ were attributed to the elongation vibrations of (C=O) in uronic monosaccharides [36]. Asymmetric vibrations of elongation within O-S-O were revealed at 1222 and 1223 cm^−1^ indicating the presence of sulfate esters [37], whilst the elongation of sulfur dioxide (O=S=O) could be indicated by the signals at 1027 and at 1013 cm^−1^ for BBF (Figure 2A) and FSF (Figure 2B), respectively [37]. In addition, sulfate groups linked to C4 of fucosyl units seem to be revealed at 836 and 833 cm^−1^ characteristics bands of (C4-O-S) elongation [38]. However, the binding of sulfate groups with galactose residues was indicated by the absorption bands at 577 and 479 cm^−1^ [37].

### 2.3. Effect of Fucoidans (FSF and BBF) on the Natural Defence of Date Palm Roots

#### 2.3.1. Phenylalanine Amonia-Lyase (PAL) Activity

**Figure 3:**
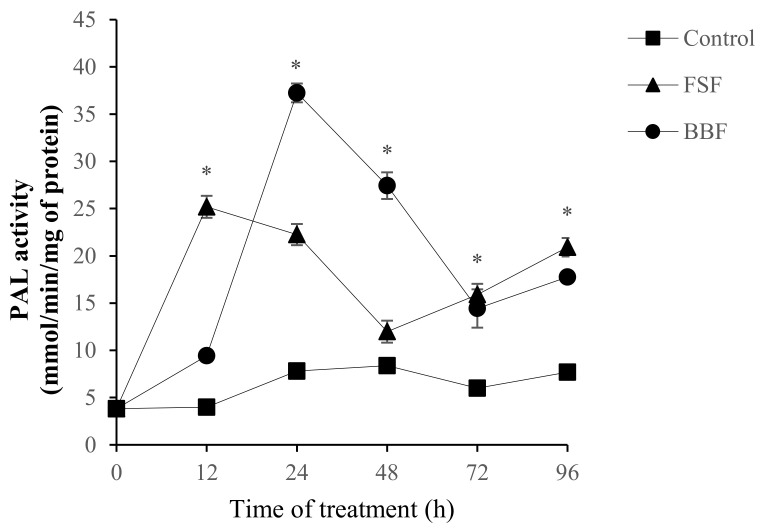
Induction of phenylalanine ammonia-lyase (PAL) activity in date palm roots treated with sulfated polysaccharides of *F. spiralis* (FSF) and *B. bifurcata* (BBF). Based on Tukey’s test at 12 h, 24 h, 72 h and 96 h * Control vs. FSF: *p* < 0.05, at 24 h, 48 h, 72 h and 96 h * Control vs. BBF: *p* < 0.05.

Given the involvement of the phenolic metabolism in the natural defenses in date palm roots against Foa [17], the mobilization of the phenlypropanoids pathway was demonstrated by studying PAL activity, as the main enzyme of this metabolic pathway. As shown in Figure 3, PAL activity was induced by both *F. spiralis* (FSF) and *B. bifurcata* (BBF) fucoidans. A total of 12 h of FSF treatment were sufficient to significantly increase PAL activity compared to the control treatment (*p* < 0.05). This increase stayed significantly different from control plants over 24 h. A second narrower peak was obtained at 96 h, this could be explained by the elicitor solutions (fucoidans) remained in permanent contact with the roots for the duration of the experiment (4 days), leading to a second wave of induction of PAL activity. The BBF treatment intensely and significantly increased PAL activity after 24 h of treatment, 4.8 times higher than the response noted in control plants (*p* < 0.05).

#### 2.3.2. Total Phenolic Compounds Content

**Figure 4:**
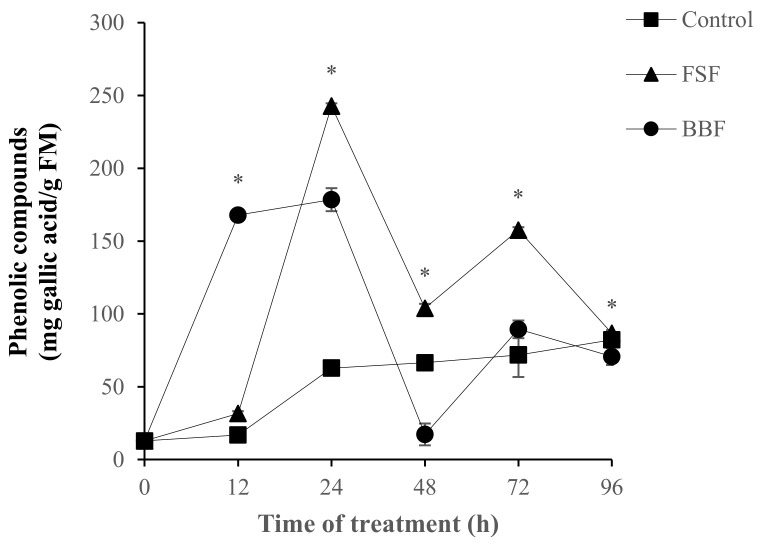
Effect of *F. spiralis* (FSF) and *B. bifurcata* (BBF) fucoidans on the accumulation of phenolic compounds in date palm roots. Means values ± SE. Based on Tukey’s test at 12 h and 24 h * Control vs. BBF: *p* < 0.05, at 24 h, 48 h and 72 h * Control vs. FSF: *p* < 0.05.

The elicitor effect of the sulfated polysaccharides studied (FSF and BBF) on phenolic metabolism was also approached by the accumulation of total phenols in treated roots. The accumulation of phenolic compounds following FSF and BBF treatments was presented in Figure 4. Roots elicitation by FSF caused a significant (*p* < 0.05) and intense accumulation of phenolic compounds after 24 h compared to the control treatment (Figure 4). Second narrower accumulation of phenols was approved after 72 h of FSF treatment, this could be explained by the induction of PAL activity at the same time. However, a precocious and significant (*p* < 0.05) accumulation of these compounds was manifested just 12 h after following BBF treatment, this level of phenolic compounds remained higher than control plants during 24 h before decreasing at 48 h.

#### 2.3.3. Accumulation of Lignin Content

**Figure 5:**
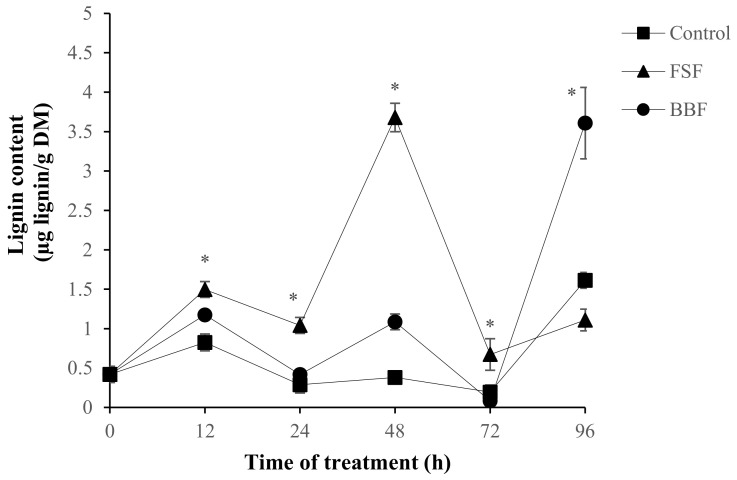
Effect of sulfated polysaccharides from *F. spiralis* (FSF) and *B. bifurcata* (BBF) on the accumulation of lignin in date palm roots. Means values ± SE. Based on Tukey’s test at 12 h, 24 h, 48 h and 72 h * Control vs. FSF: *p* < 0.05, at 12 h, 48 h and 96 h * Control vs. BBF: *p* < 0.05.

Different trend of the lignin deposition in treated roots was exhibited following elicitation by FSF and BBF (Figure 5). The level of this metabolite increased slightly after 12 h then greatly increased (*p* < 0.05) after 48 h of FSF treatment. This response was expected since lignin is a phenols polymer whose maximum accumulation was obtained after 24 h with FSF (Figure 4). On the other hand, with BBF treatment (Figure 5), the lignin contents undergo a weak increase after 24 h and 48 h of elicitation while after 96 h a higher increase was noted, this could be explained by the possibility of the polymerization of phenolic compounds increased after 72 h of BBF treatment. The highest content of lignin was obtained at 48 h in response to FSF and after 96 h of BBF treatment, it is three times higher than that obtained in control roots.

The crude fucoidans from *F. spiralis* (FSF) and *B. bifurcata* (BBF) were extracted and structurally characterized; the results obtained show a significant proportion of L-fucose in FSF and BBF with a higher degree of sulfation (45 and 49% *w*/*w* respectively). A biological test showed that the crude fucoidans (FSF and BBF) exhibit an eliciting effect of the defence mechanisms in date palm roots. These mechanisms were initiated by the induction of PAL activity. FSF and BBF expressed a slightly differential effect on PAL activity, which could be due to the difference in structure between the two fucoidans. Indeed, the structural characterization revealed differences in sulfate proportions within FSF and BBF, suggesting a difference in FSF and BBF affinity to membrane receptors. The perception and recognition of elicitors from pathogens, plants and algae called damage- or pathogen-associated molecular pattern molecules (DAMPs, PAMPs) by Pattern Recognition Receptors (PRRs) in plants induces a signalling cascade in the host cell through their cytosolic domains leading to the induction of defence mechanisms in the host plant [39]. This could be the cause of the early induction of PAL activity after treatment with FSF compared to BBF. In addition, following the induction of PAL activity, the phenolic metabolites were accumulated in the treated roots, as well as lignin deposition. Phenols and lignin are among the most involved defence elements during date palm–Foa interactions [15,16,17,18,19,20]. The results obtained are similar to the reaction observed in tobacco plants pretreated with fucoidans and sulfated oligofucoidans, in which PAL activity was also induced [14]. In addition, such tobaccos demonstrate an increase in the activity of lipoxygenase (LOX) and Pathogenesis-related protein (PR), as well as transient defence reactions such as acidification of the cytoplasm and accumulation of H_2_O_2_ [14]. Based on this, it is possible to assume similar effects in the case of date palm root response to FSF and BBF, including also the induction of glutathione-S-transferase (GST) activity, as shown for tobacco [15]. In addition, a fucoidans pre-treatment of carrot leaves protects them against *Alternaria radicina* and *Botrytis cinerea* attacks by stimulating the accumulation of phenolic compounds and by inducing peroxidase (POD) and polyphenol oxidase (PPO) activities [40]. Thus, fucoidans-treated date palm roots, besides phenolic compounds accumulation, may also activate the POD and PPO enzymes. Otherwise, we have shown in recent work, that alginates extracted from the same brown algae *F. spiralis* and *B. bifurcata* stimulate the natural defenses of date palm in the same way as fucoidans, but the latter seem to be more active at low concentrations (0.5 g/L) compared to 1 g/L of alginates [41]. This could be explained by the structural difference between alginates and fucoidans, in particular the presence of sulfated groups and the degree of sulfation. The structure–function relationship governing the induction of plant defence mechanisms in response to fucoidans stile less elucidated. However it was reported that sulfation of polysaccharides alters their affinity for receptors located in cell walls [42]. In addition, the desulfation of sulfated polysaccharides reduces or eliminates their eliciting effect on natural defenses in tomatoes [43], whereas oligo-carrageenans (λ) with a higher degree of sulfation reduce the impact of various viral, bacterial and fungal diseases [44]. Furthermore a sulfated ulvan and oligo-ulvans improve the induction of PAL activity and therefore the accumulation of the phenols in tomato leaves [43], apple fruit [3] and olive tree [45]. Numerous works on the other biological activities of fucoidans relate their effectiveness to their structural characteristics, notably sulfation and molecular weight. It has been widely documented that fucoidans owe their broad spectrum of biological activities to their sulfated nature and molecular weights [6,27,28,46,47]. Based on previous work, fucoidans with a molecular weight (Mw) between 10 and 300 × 10^3^ g/mole showed significant anticoagulant activity than those with higher Mw > 850 × 10^3^ g/mole [48,49,50]. Likewise, immune-regulation activities increased with low molecular weight fucoidans of *Laminaria japonica* [51]. It has been shown also that the immune-regulatory potential of fucoidan was influenced by the sulfate group as well as the acetyl one [52]. In short, the biological efficiency of fucoidans was modulated by several parameters, in particular the proportion of SO_4_^2−^ groups and their position, the molecular weight, the acetylation degree and monosaccharides composition [6,53].

## 3. Conclusions

The crude fucoidans from the brown algae, *F. spiralis* and *B. bifurcata* tested on the date palm roots, display potential elicitor activity on the phenolic metabolism due to the induction of PAL activity. Thus, leading to mobilisation of the phenlypropanoids pathway and to the accumulation of phenolic compounds and lignin. The elicitor effect of studied fucoidans was related to their highly sulfated structures with a small molecular weight (*M*_W_). In addition, the simple and innovative elicitation model adopted in this paper highlighted the elicitor effect of fucoidans without stressing the roots, which could be applied in the field. These results open prospects for the formulation of a biological product, leading to a preventive control of date palm Bayoud disease.

## 4. Materials and Methods

### 4.1. Extraction, Purification and Chemical Analysis of Fucoidans (FSF and BBF)

Brown algae *B. bifurcata* and *F. spiralis* were harvested on the at El Jadida city (Morocco) in December 2017. The extraction and separation of sulfated polysaccharides were performed according to Ermakova et al. [54]. Samples of 25 g of each algae species were depigmented with formaldehyde 2% and then dried for 12 h at ambient temperature. Dried powders were then treated twice with HCl 0.1 M solution during 2 h (at 60 °C, 450 rpm). After centrifugation for 20 min at 5000 rpm, the recovered supernatants were neutralized to pH 7.5. Crude fucoidans were obtained with thrice ethanol 96% (3 *v*/*v*) precipitation and then freeze-dried to *B. bifurcata* and *F. spiralis* crude fucoidans powders (BBF and FSF, respectively). All chemical analysis of FSF and BBF was performed using colorimetric assays as described in previous paper [41].

### 4.2. GC-MS Analysis of FSF and BBF

Prior to GC-MS analysis, samples of 15 mg of BBF and FSF were hydrolyzed using Trifluoroacetic acid (TFA) under 120 °C for 90 min. Monosaccharides generated by this acid hydrolysis were then treated with N,O-Bis (trimethylsilyl) trifluoroacetamide (BSTFA) with 1% Trimethylchlorosilane (TMCS) according to Pierre et al.’s method [55,56]. After evaporation, monosaccharides constituting the fucoidans (FSF and BBF) as well as the standards were injected in GC-MS at 10 g/L of dichloromethne.

### 4.3. ATR-FTIR Spectroscopy

Infrared analysis of FSF and BBF was carried out using the Attenuated Total Reflectance (ATR) technique by a VERTEX 70 FTIR system. Spectra were obtained after 50 scans in a 500–400 cm^−1^ wave range.

### 4.4. ^1^H NMR Spectroscopy Analysis

Twenty mg of crude fucoidans was prepared thrice in 0.5 mL of D_2_O. With a 400 MHz Bruker AVANCE spectrometer, the ^1^H NMR spectroscopy analysis was carried out at 60 °C.

### 4.5. Elicitation Test

Roots of three-month-old date palm plants (greenhouse model) were soaked in fucoidan solutions (FSF and BBF) at a concentration of 0.5 g/L and pH 6.5 over 4 days. Fucoidans-treated roots were compared to distilled water-treated roots as a control treatment. After 12 h and then every 24 h, biochemical assays of phenylalanine ammonialyase (PAL) activity, phenolic compounds and lignin content in treated roots were performed. Data were reported as the means values of 3 replicates, each replicate containing 3 plants.

### 4.6. Phenylalanine Ammonialyase (PAL) Activity

PAL activity was determined according to the method described by Liu et al. [57] with slight modifications. Enzyme extract was prepared at 4 °C, with 250 mg of crushed date palm roots in 3 mL of borate buffer (100 mM, pH 8.8, 4 °C) with EDTA (1 mM) and 5% (*w*/*v*) of insoluble polyvinyl polypyrrolidone (PVPP). The enzyme extract was recovered after centrifugation for 30 min and at 10,000× *g*. The reaction mixture of PAL activity assay composed of enzymatic extract (600 µL), l-phenylalanine at 20 mM (250 μL) and borate buffer (1 mL). An amount of 100 µL HCl was added after incubation (1 h, 30 °C). The results were obtained at 920 nm. The Bradford method [58] was then used to quantify the total protein in the enzyme extract.

### 4.7. Phenolic Compounds

The hydromethanolic phenolic extracts obtained according to Hagen et al.’s [59] method were then purified using the protocol described in previous paper [41]. Total phenolic determination was performed following the Folin–Ciocalteu method [60].

### 4.8. Extraction and Spectrophotometric Assay of Lignin Content

The lignin was extracted using the Bruce and West [61] protocol, with some modifications, in 0.5 mL of ethanol 90% (*v*/*v*), were grounded 500 mg of treated roots. Pellet obtained after centrifugation at 10,000× *g* for 20 min and at 4 °C was dried for 12 h at 35 °C. Samples of 25 mg of the dried residue were treated with 0.5 mL of thioglycolic acid and 1.25 mL of HCl solution at 2 M. The mixture was heated during 8 h at 100 °C. After cooling and centrifugation, 2.5 mL of NaOH was added to the pellet. The supernatant recovered after stirring for 18 h at 25 °C, was centrifuged (10,000× *g*, 20 min, 4 °C) treated with 0.5 mL of pure HCl, to precipitate lignin thioglycolic acid after incubation over 4 h at 4 °C. The absorbance at 280 nm was measured in 500 µL of NaOH. The lignin content was expressed in µg of lignin thioglycolic acid/g Dry Matter (DM).

### 4.9. Statistical Analysis

PAL activity, polyphenols and lignin contents results were tested by ANOVA analysis in SPSS software Version 20.0 using Tukey’s test. The difference between treatments is significant at *p* < 0.05.

## Figures and Tables

**Table 1:** Chemical composition and yield of *F. spiralis* (FSF) and *B. bifurcata* (BBF) crude fucoidans.

Analytical Data (%, *w*/*w*)	FSF	BBF
Yield ^a^	7.9	1.9
Neutral sugar ^b^	51.16	45.23
Uronic acids ^b^	14.68	21.79
Sulfates ^b^	49.53	45.49
Protein ^b^	Traces	Traces
M_w_ (g/mol) ^c^	20 × 10^3^	14 × 10^3^

**^a^**Expressed on the weight of dry depigmented algae. **^b^** Expressed on the weight of dry fucoidans. **^c^** Molecular weight by High Performance Size Exclusion Chromatography (HPSEC) analysis.

**Table 2:** Monosaccharide composition of *F. spiralis* (FSF) and *B. bifurcata* (BBF) crude fucoidans.

Monosaccharides ^a^ (% mol)	FSF	BBF
Fucose	63.98	90.68
Galactose	20.00	6.19
Glucose	8.00	nd
Mannose	7.99	1.65
Rhamnose	nd	1.46
Fuc_p_/Gal_p_ ratio	3.2	14.7

**^a^** Monosaccharides composition by GC-MS analysis, expressed as molar % of the total identified peaks based on the weight of dry fucoidans.
